# Three-Dimensional Finite Element Analysis of Anterior Two-Unit Cantilever Resin-Bonded Fixed Dental Prostheses

**DOI:** 10.1155/2015/864389

**Published:** 2015-03-24

**Authors:** Filip Keulemans, Akikazu Shinya, Lippo V. J. Lassila, Pekka K. Vallittu, Cornelis J. Kleverlaan, Albert J. Feilzer, Roeland J. G. De Moor

**Affiliations:** ^1^Department of Restorative Dentistry and Endodontology, Dental School, Ghent University Hospital, Ghent University, De Pintelaan 185/P8, 9000 Gent, Belgium; ^2^Department of Biomaterials Science, BioCity Turku Biomaterials Research Program, Institute of Dentistry, University of Turku, Lemminkäisenkatu 2, 20520 Turku, Finland; ^3^Department of Crown and Bridge, School of Life Dentistry at Tokyo, The Nippon Dental University, 1-9-20 Fujimi, Chiyoda-ku, Tokyo 102-8158, Japan; ^4^Department of Dental Materials Science, Academic Centre for Dentistry Amsterdam (ACTA), University of Amsterdam and VU University Amsterdam, Mahlerlaan 3004, 1081 LA Amsterdam, Netherlands

## Abstract

The aim of this study was to evaluate the influence of different framework materials on biomechanical behaviour of anterior two-unit cantilever resin-bonded fixed dental prostheses (RBFDPs). A three-dimensional finite element model of a two-unit cantilever RBFDP replacing a maxillary lateral incisor was created. Five framework materials were evaluated: direct fibre-reinforced composite (FRC-Z250), indirect fibre-reinforced composite (FRC-ES), gold alloy (M), glass ceramic (GC), and zirconia (ZI). Finite element analysis was performed and stress distribution was evaluated. A similar stress pattern, with stress concentrations in the connector area, was observed in RBFDPs for all materials. Maximal principal stress showed a decreasing order: ZI > M > GC > FRC-ES > FRC-Z250. The maximum displacement of RBFDPs was higher for FRC-Z250 and FRC-ES than for M, GC, and ZI. FE analysis depicted differences in location of the maximum stress at the luting cement interface between materials. For FRC-Z250 and FRC-ES, the maximum stress was located in the upper part of the proximal area of the retainer, whereas, for M, GC, and ZI, the maximum stress was located at the cervical outline of the retainer. The present study revealed differences in biomechanical behaviour between all RBFDPs. The general observation was that a RBFDP made of FRC provided a more favourable stress distribution.

## 1. Introduction

Resin-bonded fixed dental prostheses (RBFDPs) have proven to be a reliable treatment alternative for the replacement of missing teeth [1, 2] especially in cases where conservation of tooth tissue is needed and limited financial resources are available. According to a recent systematic review, RBFDPs exhibit an estimated survival rate of 87.7% (95% confidence interval: 81.6%–91.9%) after 5 years [3]. Notwithstanding their good clinical performance, the most frequent complication was debonding, with a reported cumulative debonding rate of 19.2% (95% CI: 13.8–26.3%) after 5 years [3].

The use of more extensive preparation of the abutment teeth, including palatal or lingual coverage with 180-degree wraparound, chamfer, cingulum rests, and proximal guide planes and grooves, is a way to improve the retention of RBFDPs [4]. Another way to minimize debonding is to design RBFDPs as a two-unit cantilever. Several clinical studies of the last decade have demonstrated that two-unit cantilever RBFDPs performed as well as or even better than their three-unit fixed-fixed counterparts [5–11]. Elimination of interfacial stresses, induced by a combination of dynamic tooth contacts and differential movements of the abutment teeth, is the most widely accepted explanation for their successful clinical performance [4, 12].

The framework of RBFDPs is traditionally made of metal alloys, but their poor aesthetics and the growing awareness towards possible adverse health effects of dental alloys, such as Ni-, Cr-, Co-, Pd-, and Au-containing alloys [13–17], stimulated the interest in metal-free restorations. Nowadays, all-ceramic [10] and fibre-reinforced composites (FRC) [18, 19] are viable alternatives for framework fabrication of RBFDPs. Some clinical cases reported promising results for all-ceramic RBFDPs [20, 21]. In addition Kern and Sasse reported 10-year survival rates for glass-infiltrated alumina-based RBFDPs of 73.9% for three-unit fixed-fixed designs and 94.4% for two-unit cantilever designs [11]. The same authors reported a survival rate of 93.3% after 5 years for single-retainer zirconia-based RBFDPs [22]. Finally, Sailer et al. evaluated the clinical performance of single-retainer lithium disilicate glass ceramic-based RBFDPs finding a 5-year survival rate of 100% [23]. A recently published systematic review reported for FRC-FDPs a survival rate of 73.4% (95% CI: 69.4–77.4%) after 4.5 years [19]. During a 5-year multicenter clinical study FRC RBFDPs exhibited a survival rate of 64% [24]. The differences in material properties, especially elastic modulus, adhesive properties, and thermal expansion coefficient, are believed to affect the mechanical and clinical performance of RBFDPs [25]. In order to better understand the failure mechanism of two-unit cantilever RBFDPs, increased knowledge on the biomechanical behaviour of these restorations is needed.

The aim of the present study was to compare, by means of three-dimensional finite element analysis (3DFEA), the biomechanical behaviour of anterior two-unit cantilever RBFDPs made of various framework materials.

## 2. Material and Methods

### 2.1. Definition of Structures, Geometric Conditions, and Materials

**Figure 1:**
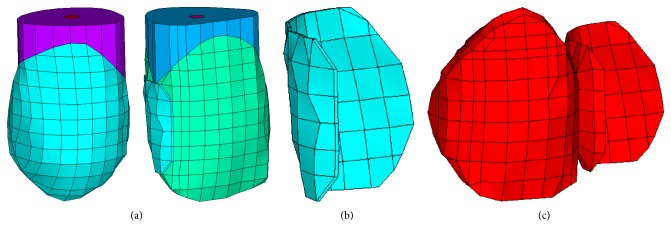
3D FE model of a cantilever two-unit RBFDP: (a) abutment and adjacent tooth, (b) cement layer, and (c) RBFDP.

A FE model representing a single tooth gap in the anterior right maxilla, consisting of a central incisor, a missing lateral incisor, and a canine (Figure 1(a)), was created. The central incisor served as the abutment tooth but was not provided with a retainer preparation. The missing lateral incisor was replaced by a two-unit cantilever RBFDP (Figure 1(c)) with a retainer on the central incisor. A wing-shaped retainer design, which enwrapped the palatal and distal surface of the abutment tooth, was selected and the pontic was shaped according a modified ridge lap design. Three-dimensional FE model of the cement layer, with a uniform thickness of 100 *μ*m, is shown in Figure 1(b). A more detailed description of the creation of the FE model was published earlier by Shinya et al. [25].

The geometry of the healthy standard tooth as abutment has been previously described [26]. Not only the natural tooth geometry but also the composition (enamel, dentine, and pulp tissue) was mimicked. Roots under the bone level, periodontal ligaments, and alveolar bone were not created.

Materials properties are derived from clinically used materials (reference brand between parentheses): hybrid particulate filler composite (PFC) for laboratory use (Estenia C&B; Kuraray medical Inc., Tokyo, Japan), hybrid PFC for chairside use (Filtek Z250; 3M ESPE, MN, USA), unidirectional FRC for laboratory use (Estenia C&B EG fiber; Kuraray medical Inc., Tokyo, Japan), unidirectional fibre-reinforced composite for direct and chairside use (EverStick C&B; StickTech Ltd., Turku, Finland), Au-Pd alloy (Olympia; J.F. Jelenko, Armork, NY, USA), lithium disilicate glass ceramic (IPS Empress 2; Ivoclar-Vivadent, Schaan, Liechtenstein), zirconia (InCeram Zirconia; Vita, Bad Säckingen, Germany), feldspathic porcelain (Creation; Klema, Meiningen, Austria), resin-based luting cement (Variolink 2; Ivoclar-Vivadent, Schaan, Liechtenstein), enamel, dentin, and pulp. The material properties, mostly obtained from existing literature, are summarised in Table 1. The materials were assumed to be isotropic, homogeneous, and linear elastic, except for FRC. The mechanical behaviour of continuous unidirectional FRC, influenced by their anisotropic (orthotropic) properties, can be described by 3 young's moduli, 3 Poisson's ratios, and 3 shear moduli [27]. Twenty-node brick element such as solid 95 in ANSYS has the anisotropic material option. The orientation of the element coordinate system was altered in such a way that it matched the fibre direction.

**Figure 2:**
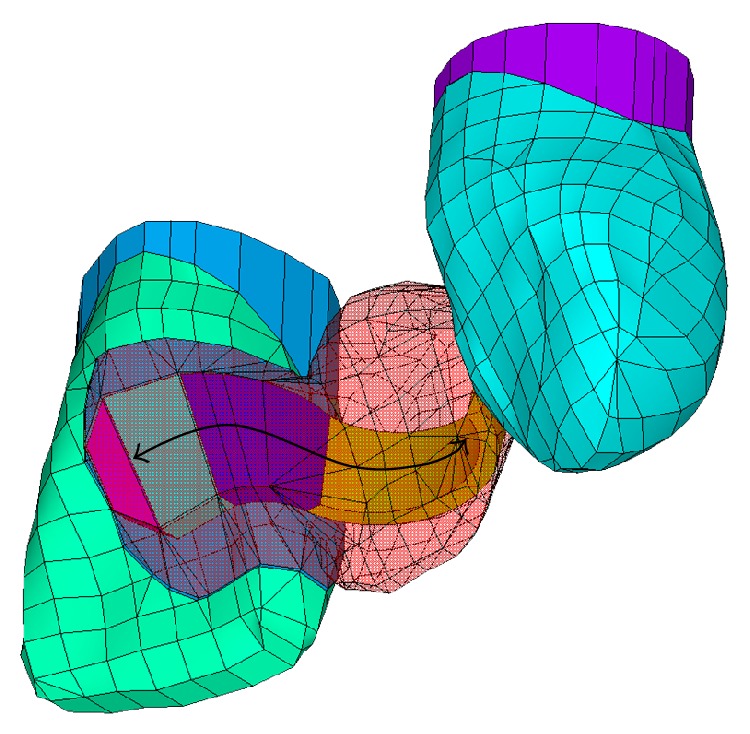
3D FE model of a two-unit cantilever FRC RBFDP: position of the FRC framework in relation to the FDP and the abutment teeth is shown. Double arrowed black line represents the fibre direction.

Five different groups with various framework materials were evaluated:

1. FRC-Z250: a FRC-FDP made of continuous unidirectional E-glass FRC framework (Figure 2) veneered with hybrid PFC for direct and chairside use;
2. FRC-ES: a FRC-FDP made of continuous unidirectional E-glass FRC framework veneered with hybrid PFC for laboratory use;
3. M: a metal-ceramic FDP made of type 3 Au-Pd alloy framework veneered with feldspathic porcelain;
4. GC: an all-ceramic FDP made of lithium disilicate glass ceramic framework veneered with feldspathic porcelain;
5. ZI: an all-ceramic FDP made of zirconia framework and veneered with feldspathic porcelain.

A FRC framework was designed with thickness of 0.6 mm and a height of 3.0 mm [28]. The three-dimensional FE model of the FRC framework and its position in relation to the RBFDP are shown in Figure 2.

### 2.2. Mesh Generation, Boundary Conditions, and Data Processing

**Figure 3:**
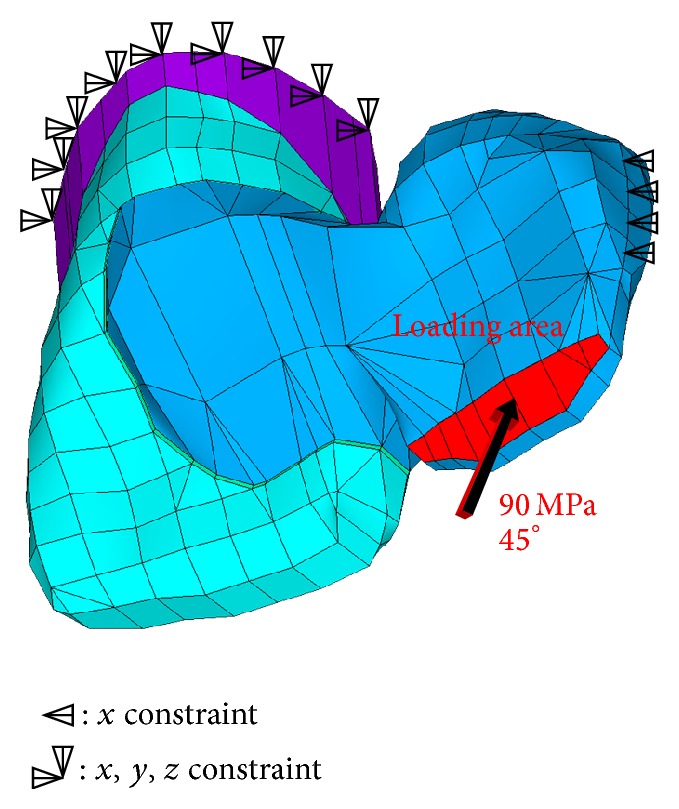
Loading and boundary conditions of a 3D FE model representing two-unit cantilever RBFDPs.

In order to avoid quantitative differences in stress value, all solid models were derived from a single mapping mesh pattern that generated 103,861 twenty-node brick element (Solid 95 in ANSYS) and 154,784 nodes. Loading and boundary conditions are depicted in Figure 3. A stress of 90 MPa was applied at a 45° angle to the incisal edge of the pontic. In the present study, the FE model was loaded by applying a stress of 90 MPa in a 45° angle to the incisal edge of the pontic tooth. An applied stress of 90 MPa to a 5.5 mm² incisal area corresponds to a load of 495 N. The applied load is significantly higher than previously reported maximum anterior mastication loads of 108–382 N [29, 30] and therefore can be regarded as the worst-case scenario. In clinical circumstances, an anterior occlusal contact more closely resembles an area than a point; for that reason it was chosen to apply the load in an area. The terminal elements of the abutment tooth were fixed in all directions, as well as the final elements of the contact area to canine in distal direction. 3DFEA was presumed to be linear static and was performed on PC workstation (Precision Work Station M90, Dell Inc., Texas, USA) using FE analysis software ANSYS 11 (ANSYS Inc.; Houston, TX, USA). The locations and magnitudes of the principal stress (MPa) and displacement (mm) were identified and used for evaluating the biomechanical behaviour. Maximum principal stress describes the highest stress and can be regarded to be a tensile stress.

## 3. Results

### 3.1. Stresses in the FDP (Figure 4)

Differences in maximum principal stress were observed (Table 2) and showed a decreasing order: ZI > M > GC > FRC-ES > FRC-Z250. Stress concentrations were located in the connector area, more precisely at the occlusal embrasure, for all framework materials. However, additional stress concentrations were observed at the contact area with the adjacent tooth for all framework materials and at the mesiocervical edge of the retainer for GC (20–30 MPa), M (30–40 MPa), and ZI (50–70 MPa). Stresses at the contact area with the adjacent tooth were lower for FRC-ES and FRC-Z250 (30–40 MPa) in comparison to GC (50–70 MPa), M, and ZI (>70 MPa).

### 3.2. Stresses at the Cement-Retainer Interface (Figure 5)

Differences in maximal principal stress were also observed (Table 2) at the cement-retainer interface and showed a decreasing order: ZI > M > GC > FRC-ES > FRC-Z250. However, their location differed and was observed in the upper part of the proximal area for FRC-Z250 and FRC-ES, while they were located in a semicircular way around the connector and at the cervical edge of the retainer for M, GC, and ZI.

### 3.3. Stresses in the Cement Layer (Figure 6)

FEA revealed (Table 2) only slight differences in maximal principal stress and showed a decreasing order: FRC-Z250 > ZI > FRC-ES > M > GC. They were located in a different area of the cement layer. Highest stress concentrations were located in the upper part of the proximal area for FRC-Z250 and FRC-ES, while they were located at the cervical margin for M, GC, and ZI.

### 3.4. Stresses on the Abutment Tooth (Figure 7)

On the abutment tooth only slight differences in maximal principal stress were observed (Table 2). Highest value was 34.9 MPa for FRC-Z250 and the lowest value was 30.9 MPa for FRC-ES. Once again, their location showed some differences. Highest stress concentrations for FRC-Z250 and FRC-ES were observed at the upper middle part of the proximal area and were surrounded by a large area of stress concentration (17–31 MPa) that extended towards the palatocervical area, while they were located in a small area of the palatocervical area of the abutment tooth for M, GC, and ZI.

### 3.5. Displacement (Table 2)

Differences in maximum displacement were observed in the pontic part of the RBFDP between the different materials. Higher displacement of the RBFDP was encountered with FRC-Z250 and FRC-ES and then with M, GC, and ZI. Although, the maximum displacement at the cement-retainer interface, cement layer, and abutment tooth revealed the same trend as those for RBFDPs, a difference of 0.001 mm between highest and lowest value could not be regarded as clinically relevant.

## 4. Discussion

A static fracture strength test, during which a FDP is vertically loaded till failure, is the most common way to evaluate the mechanical behaviour of FDPs in laboratory conditions [31]. The drawbacks of this approach, such as the difficulty in fabricating uniform FDPs in terms of shape and dimensions, are reckoned by researchers familiar with it. Although, FEA can be regarded as a relatively easy and inexpensive way to evaluate the mechanical behaviour of complex structures, some limitations should be acknowledged. Some of these limitations can be drawn back to the simplifications made to the finite element models, for example, tooth model without roots, periodontal ligament [32] and, bone, and the assumptions made related to their material properties [33]. The latter were illustrated by the fact that all materials, except FRC, were assumed to be isotropic, homogenous, and linear elastic, despite the anisotropic nature of tooth tissue like dentine [34]. Therefore, one should be aware of the fact that the reported values cannot be regarded as absolute values. The main purpose of this study was to compare the biomechanical behaviour of anterior two-unit cantilever RBFDP made of different framework materials. Nevertheless, the ideal approach is to interpret the results from both FEA and mechanical testing simultaneously, which allows providing more reliable and validated data than either method alone [35]. So mechanical testing on two-unit cantilever RBFDPs in the same condition as this study could be a valuable asset.

In the present study, the FE model was loaded by applying a stress of 90 MPa in a 45° angle to the incisal edge of the pontic tooth. An applied stress of 90 MPa to a 5.5 mm² incisal area corresponds to a load of 495 N. The applied load is significantly higher than previously reported maximum anterior mastication loads of 108–382 N [29, 30] and therefore can be regarded as the worst-case scenario. In clinical circumstances, an anterior occlusal contact more closely resembles an area than a point, for that reason it was chosen to apply the load in an area.

Roots, periodontal ligament, and bone, which are responsible for physiologic tooth mobility, were not included in the FE model. Under clinical conditions, a part of the loading is transferred via the roots and the periodontal ligament into the bone. The lack of physiologic tooth mobility in the present FE model negatively influences the outcome of the FEA, in such a way the principal stress values are overestimated. The effect of tooth mobility was illustrated by Rosentritt et al., who found higher fracture strengths for anterior cantilever RBFDPs when luted to abutment teeth with high mobility [36]. Clinically, rationality to use cantilever design over fixed-fixed design is related to the teeth mobility. If teeth with increased mobility are involved, risk for debonding of fixed-fixed RBFPD from one end is relatively high. A debonded retainer may result in secondary caries that often is not diagnosed in time [2, 4, 7].

The present FEA revealed differences in biomechanical behaviour between RBFDPs made of different framework materials. Although the location of the stress concentration, observed at the FDP level, was identical for all framework materials, the values differed. The differences in displacement and principal stress can be explained by the differences in elastic modulus between framework materials. RBFDPs made of materials with higher stiffness suffered less displacement, but higher principal stress, than those made of less stiff materials, which can be illustrated by comparison of zirconia and chairside FRC. Zirconia exhibits an elastic modulus of 205 GPa and showed 0.017 mm displacement and 239.6 MPa maximum principal stress in comparison to 0.048 mm and 156.9 MPa by the chairside FRC with an elastic modulus between 11 GPa (chairside hybrid composite) and 46 GPa (FRC). The highest maximum principal stress was located at the occlusal embrasure of the connector. It has to be noticed that the connector in our FE model was designed with a sharp embrasure and that stresses in this location can be significantly decreased by changing the connector design [37] and its radius of curvature [37, 38]. Recently, Plengsombut et al. confirmed this finding by revealing a significant lower fracture strength for specimens with a round connector in comparison to those with a sharp connector [39].

**Figure 5:**
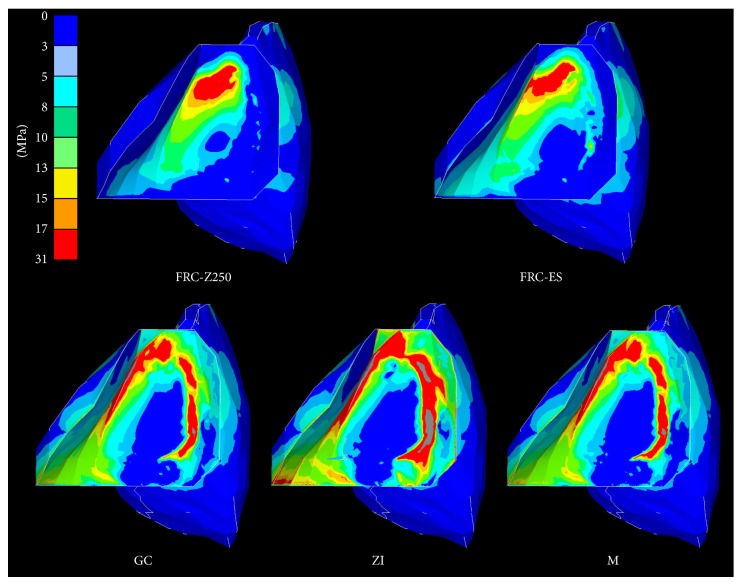
Principal stress distribution at the cement-retainer interface for two-unit cantilever RBFDPs of various framework materials.

A similar situation with regard to stress values was found at the level of cement-retainer interface. Far more interesting were the differences in location between FRC on one hand and M, GC, and ZI on the other hand (Figure 5). A possible explanation is the difference in design between both groups of FDPs. In a FRC-FDP the stiffer fibres acts as a stress dissipater and transfers the stress from the pontic to the central part of the retainer. On the contrary, with FDPs made of a more stiff and uniform framework (M, GC and ZI) the stress is transferred more uniformly through the FDP to an area around the connector and towards the cervical margin of the retainer. Debonding of the FDPs due to early failure of the adhesive interface between retainer and cement layer is likely to be caused by such unfavourable stress location in combination with direct exposure to the oral environment. In particular zirconia, known for its weak adhesion to resin luting cements [40–42], will be prone to adhesive failure.

**Figure 6:**
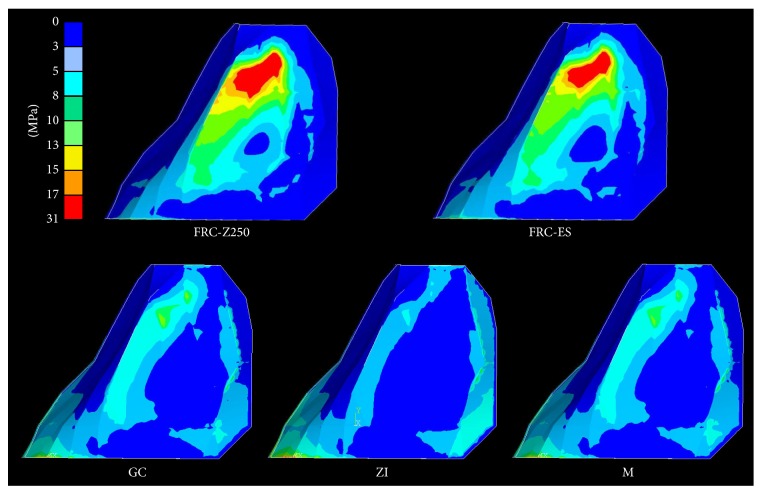
Principal stress distribution within the cement layer for two-unit cantilever RBFDPs of various framework materials.

At the level of the cement layer there was only a slight difference in maximum principal stress values, but as expected the differences in location, as seen at the cement-retainer interface, between FRC on one hand and M, GC, and ZI on the other hand (Figure 6) became more pronounced. It is noteworthy that the cement layer, in the case of M, GC, and ZI, is able to absorb the stresses in the area surrounding the connector and to dissipate them towards the cervical outline. Stress transfer towards unfavourable locations can result in premature failure of the cement layer.

**Figure 7:**
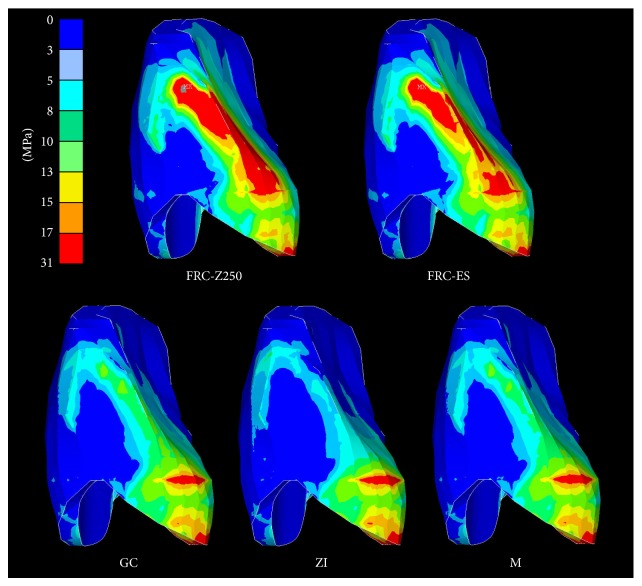
Principal stress distribution at the abutment tooth for two-unit cantilever RBFDPs of various framework materials.

The difference in maximum principal stress value between different framework materials was even lower at the level of the abutment tooth. However, the location of the stress concentration, as depicted in Figure 7, was different. Adhesive failure at the enamel-cement interface is not very likely to occur, as enamel bonding is a reliable procedure with reported values for resin luting cements, like Variolink 2, of 49.3 MPa [43].

Based on the results of this study the predominant failure mode of two-unit cantilever RBFDPs for each framework material might be predicted. Although acceptable bond strength to resin luting cements can be achieved by glass ceramics, their low strength could make them susceptible to connector fracture and therefore probably less suitable for the fabrication of anterior two-unit cantilever RBFDPs. On the contrary, the only clinical study published on cantilever glass ceramic RBFDPs reported 100% survival after 6-year concluding [23]. Nevertheless, in this study some minor chippings at the pontic were described [23]. There are more studies available on cantilever alumina RBFDPs [11, 44, 45]. These cantilever alumina RBFDPs exhibited a 10-year survival rate of 94.4% [11]. During their study only one cantilever RBFDP was lost due to fracture of the connector. Koutayas et al. reported connector fracture as the predominant fatigue failure of cantilever alumina RBFDPs [44, 45]. Since glass ceramics exhibit flexural strength of 252 MPa [46], which is inferior to the flexural strength of alumina (429 MPa) [47], and their bond strength to resin luting cements is superior to that of alumina [48], more fractures would be expected with cantilever glass ceramic RBFDPs. A possible explanation is the fact that alumina-based RBFDPs are made of an alumina core veneered with feldspathic porcelain, while lithium disilicate glass ceramic-based RBFDPS can be made from monolithic lithium disilicate. It is known that monolithic ceramic restorations exhibit higher fracture strength than bilayered or veneered ceramic restorations [49, 50].

Although FRC RBFDPs seem to be more promising as they exhibit good bond strength to resin luting cement, connector fracture seems to be the failure mode to be expected. Clinical [51] and* in vitro* [52, 53] findings on FRC RBFDPs also confirm this prediction. Connector fracture in all-ceramic RBFDs results in immediate loss of the pontic resulting in an acute aesthetic problem, while in case of FRC RBFDPs the glass fibres keeps the pontic in place. Nevertheless, they are at the moment only suitable as low cost temporary alternative due to the low strength of the veneering composite. Further improvements can be expected from modified framework designs [54, 55] and improved resin composites [56].

Zirconia and metal RBFDPs are suspected to fail most likely because of debonding. A multitude of clinical research on cantilever metal RBFDPs corroborates this prediction [6, 8, 9], since debonding was reported as the major reason of failure. A metal alloy exhibits plasticity, which can explain this mode of failure. Zirconia, regardless of its high strength, does not seem to be the ideal material for cantilever RBFDPs, due to the unfavourable stress distribution and low bond strength to resin luting cement leading to premature debonding. Only a limited amount of* in vitro* and* in vivo* studies on zirconia RBFDPs is available.* In vitro* studies have shown that minimal invasive cantilever zirconia-based RBFDPs subjected to fatigue loading predominantly failed due to debonding [36, 57]. However, the same studies showed a decrease in percentage of debonding in favour of retainer fractures, when a more retentive retainer design was used. Although, one should be aware that the high stress concentration at the mesiocervical edge of the retainer indicates (Figure 5) that retainer fracture in those studies is most probably the result of partial debonding. Due to partial debonding more complex torque and bending forces act on the retainer, which results in retainer fracture. The only clinical study on cantilever zirconia-based RBFDPs also reported debonding as major failure [22]. Recent improvement of the adhesive performance of zirconia by selective infiltration etching increased the bond strength to Panavia F2.0 up to 49.8 MPa [42]. The achievement of a strong and durable bond with zirconia-based materials makes it the most promising alternative to metal-based anterior two-unit cantilever RBFDPs.

## 5. Conclusions

Within the limitations of this study, 3DFEA revealed differences in biomechanical behaviour between RBFDPs made of different framework materials.

1. The general observation was that a RBFDP made of FRC provided a more evenly distributed stress pattern from loading area towards abutment tooth.
2. Maximum principal stress was located at the occlusal embrasure of the connector for all framework materials: highest value was found for ZI, while the lowest was found for FRC-Z250.
3. Advanced stress analyses suggest a possible difference in predominant failure mode: connector fracture for FRC- and glass ceramic-based RBFDPs and debonding for metal- and zirconia-based RBFDPs.
4. A stress concentration was found at the contact area with the adjacent tooth, indicating that the applied load is partially transferred to the adjacent tooth.

## Figures and Tables

**Figure 4:**
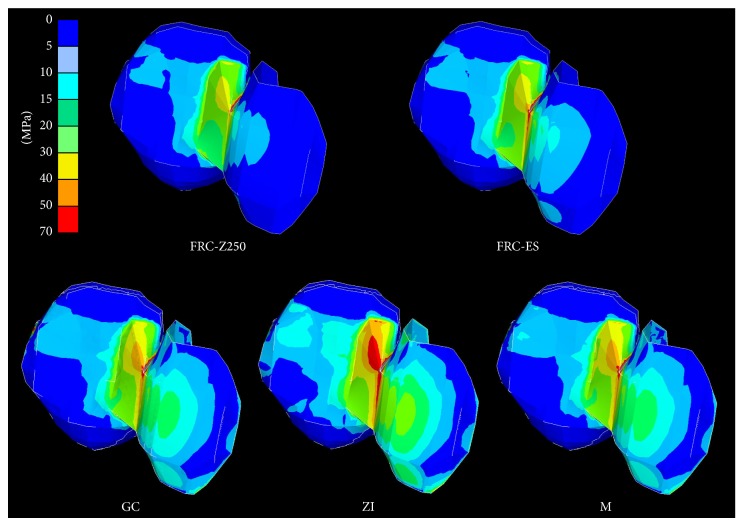
Principal stress distribution within two-unit cantilever RBFDPs of various framework materials.

**Table 1:** Elastic properties of the materials used in the finite element model.

	*E* modulus (GPa)	Poisson's ratio	Shear modulus (MPa)	Reference
Enamel	80.0	0.30	—	[58]
Dentin	17.6	0.25	—	[59]
Pulp	0.002	0.45	—	[60, 61]
Resin luting cement	8.3	0.24	—	[62]
Chairside PFC	11.5	0.31	—	[63, 64]
Laboratory PFC	22.0	0.27	—	[27]
Chairside FRC				a
Longitudinal (*X*)	46.0	0.39	16.5	
Transverse (*Y*, *Z*)	7.0	0.29	2.7	
Laboratory FRC				[27]
Longitudinal (*X*)	39.0	0.35	14.0	
Transverse (*Y*, *Z*)	12.0	0.11	5.4	
Lithium disilicate glass ceramic	96.0	0.25	—	[62]
Zirconia	205	0.22	—	[62]
Au-Pd alloy	103	0.33	—	[65, 66]

a: data obtained by StickTech Ltd. (Turku, Finland).

**Table 2:** Maximum and minimum principal stress (MPa) and displacement (mm) for two-unit cantilever RBFDPs of various framework materials.

	FDP			Cement-retainer interface			Cement layer			Abutment tooth		
	Max.	Min.	Disp.	Max.	Min.	Disp.	Max.	Min.	Disp.	Max.	Min.	Disp.
FRC-Z250	156.9	−56.2	0.048	17.5	−5.3	0.010	31.3	−7.1	0.010	34.9	−7.6	0.010
FRC-ES	177.1	−67.2	0.035	23.9	−9.7	0.010	27.3	−7.1	0.010	30.9	−9.8	0.010
GC	178.4	−116.3	0.019	32.7	−42.5	0.009	23.7	−4.1	0.009	31.4	−4.8	0.009
ZI	239.6	−154.3	0.017	60.8	−75.3	0.009	27.5	−3.3	0.009	31.7	−7.2	0.009
M	197.1	−149.9	0.019	36.1	−45.8	0.009	24.5	−3.7	0.009	31.9	−5.0	0.009
